# Supplementary material for: Characterization of the Gut Microbiota in Individuals with Overweight or Obesity during a Real-World Weight Loss Dietary Program: A Focus on the Bacteroides 2 Enterotype
Source: Biomedicines. 2021 Dec 22;10(1):16. doi: 10.3390/biomedicines10010016 (PMC8772804; doi:10.3390/biomedicines10010016)
Supplement: Supplementary file 1 [file biomedicines-10-00016-s001.zip › Supplemental Figures and Table/Supplemental Figure S2.pdf]

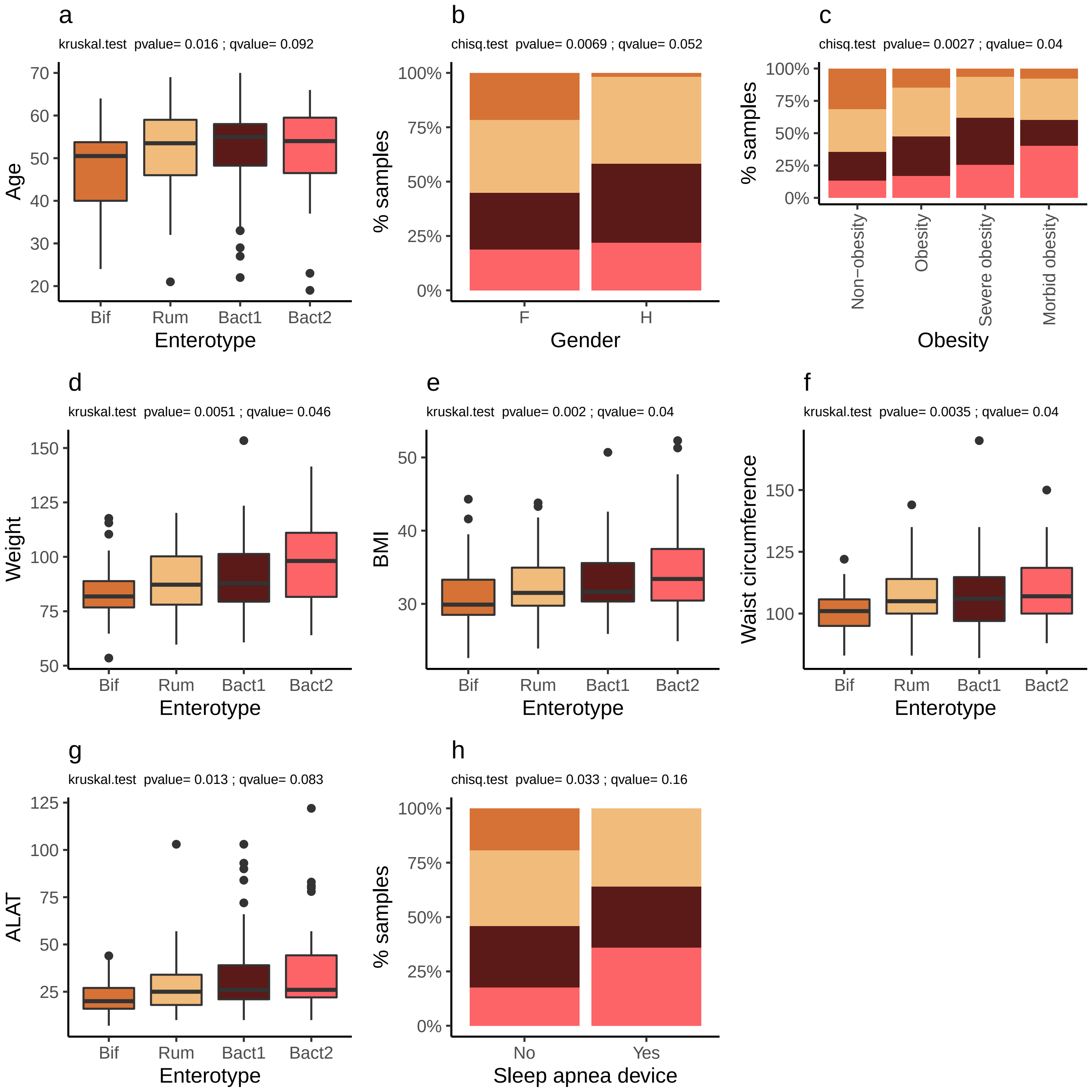

a
kruskal.test pvalue= 0.016 ; qvalue= 0.092
Age
Enterotype
Bif
Rum
Bact1
Bact2
b
chisq.test pvalue= 0.0069 ; qvalue= 0.052
% samples
Gender
F
H
c
chisq.test pvalue= 0.0027 ; qvalue= 0.04
% samples
Obesity
Non−obesity
Obesity
Severe obesity
Morbid obesity
d
kruskal.test pvalue= 0.0051 ; qvalue= 0.046
Weight
Enterotype
e
kruskal.test pvalue= 0.002 ; qvalue= 0.04
BMI
Enterotype
f
kruskal.test pvalue= 0.0035 ; qvalue= 0.04
Waist circumference
Enterotype
g
kruskal.test pvalue= 0.013 ; qvalue= 0.083
ALAT
Enterotype
h
chisq.test pvalue= 0.033 ; qvalue= 0.16
% samples
Sleep apnea device
No
Yes
