# Supplementary material for: Characterization of the Gut Microbiota in Individuals with Overweight or Obesity during a Real-World Weight Loss Dietary Program: A Focus on the Bacteroides 2 Enterotype
Source: Biomedicines. 2021 Dec 22;10(1):16. doi: 10.3390/biomedicines10010016 (PMC8772804; doi:10.3390/biomedicines10010016)
Supplement: Supplementary file 1 [file biomedicines-10-00016-s001.zip › Supplemental Figures and Table/Supplementary Table S1.pdf]

| Clinical parameters | no LMP<br>Before weight loss | no LMP<br>After weight loss (-10%) | *p*-value | LMP<br>Before weight loss | LMP<br>After weight loss (-1[illegible] |
|---|---|---|---|---|---|
| Sample size, n(%) | 47 (28.8) | - | - | 116 (71.2) | - |
| Age (years) | 52.1 (9.1) | - | - | 50.3 (10.4) | - |
| Sex (male), n (%) | 14 (29.8) | - | - | 17 (14.7) | - |
| Height (cm) | 166.7 (9.0) | - | - | 164.9 (7.3) | - |
| SAS (Yes), n (%) | 7 (14.9) | - | - | 18 (15.5) | - |
| **Adiposity markers** | | | | | |
| Weight (kg) | 91.8 (18.2) | 81.6 (16.2) | **** | 89.8 (15.3) | 80.0 (13.7) |
| BMI (kg/m$^2$) | 32.9 (4.8) | 29.3 (4.3) | **** | 33.0 (4.9) | 29.4 (4.4) |
| Waist cirumferance (cm) | 107.1 (13.8) | 95.8 (11.5) | **** | 106.8 (12.0) | 96.5 (11.8) |
| Fat mass (%) | 40.1 (4.0) | 37.0 (4.7) | **** | 41.1 (4.5) | 38.0 (5.1) |
| Fat-free mass (%) | 31.1 (6.4) | 32.1 (6.5) | **** | 33.0 (8.9) | 33.7 (8.0) |
| Calculated Basal metabolism (Kcal/day) | 2234 (366) | 2128 (358) | **** | 2179 (278) | 2061 (265) |
| **Plasma Glucose homeostasis** | | | | | |
| Glycemia (g/l) | 1.08 (0.25) | 1.00 (0.14) | * | 1.00 (0.16) | 0.94 (0.10) |
| Insulinemia (mIU/L) | 17.1 (10.6) | 9.0 (6.2) | *na* | 14.3 (7.2) | 9.7 (7.3) |
| HOMA IR | 5.4 (4.6) | 2.4 (2.0) | *na* | 3.9 (2.6) | 2.1 (1.9) |
| HbA1c (%) | 6.0 (0.9) | 5.6 (0.3) | * | 5.7 (0.8) | 5.6 (0.6) |
| **Plasma lipid homeostasis** | | | | | |
| Total cholesterol (nmol/L) | 5.8 (1.0) | 5.1 (1.1) | *** | 5.6 (1.1) | 5.1 (0.9) |
| Total triglycerides (nmol/L) | 1.6 (0.9) | 1.0 (0.4) | **** | 1.6 (0.8) | 1.1 (0.6) |
| HDL cholesterol (nmol/L) | 1.4 (0.4) | 1.4 (0.3) | *ns* | 1.4 (0.4) | 1.5 (0.4) |
| LDL cholesterol (nmol/L) | 3.4 (0.8) | 3.3 (0.8) | ** | 3.5 (0.9) | 3.1 (0.8) |
| **Liver Enzymes** | | | | | |
| ASAT (IU/L) | 23.9 (9.5) | 22.5 (8.6) | *ns* | 23.3 (7.7) | 21.2 (6.9) |
| ALAT (IU/L) | 32.2 (19.4) | 26.8 (14.2) | * | 28.5 (16.3) | 23.2 (10.6) |
| GGT (IU/L) | 45.7 (33.4) | 22.6 (17.2) | ** | 39.2 (41.0) | 33.7 (42.9) |
| **Other variables** | | | | | |
| Creatinine (mg/L) | 8.1 (1.5) | 8.1 (1.4) | *ns* | 8.2 (1.4) | 8.3 (1.4) |
| Uricemia (mg/L) | 54.9 (12.4) | 50.2 (11.3) | *ns* | 52.0 (16.4) | 46.9 (15.0) |
