# Supplementary figures and images for: Characterization of the Gut Microbiota in Individuals with Overweight or Obesity during a Real-World Weight Loss Dietary Program: A Focus on the Bacteroides 2 Enterotype
Source: Biomedicines. 2021 Dec 22;10(1):16. doi: 10.3390/biomedicines10010016 (PMC8772804; doi:10.3390/biomedicines10010016)

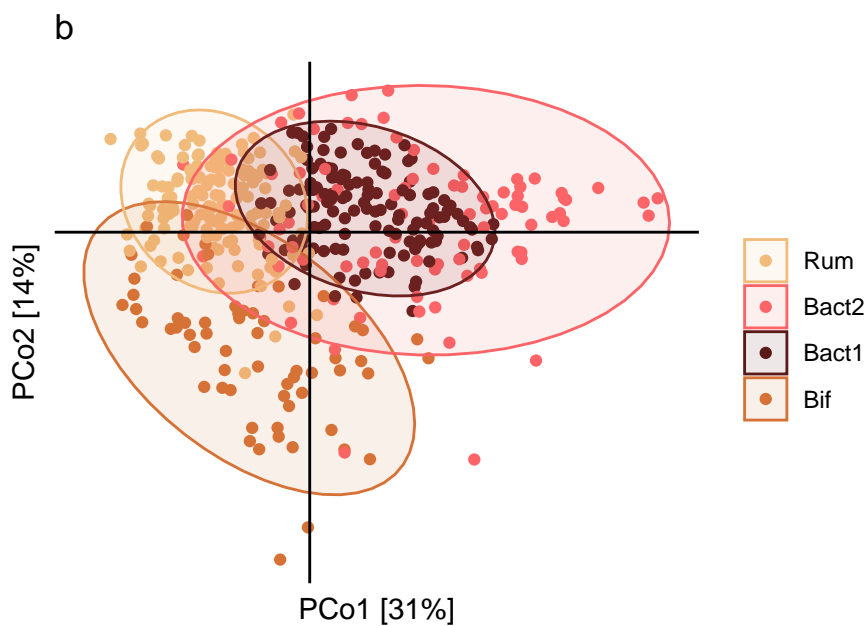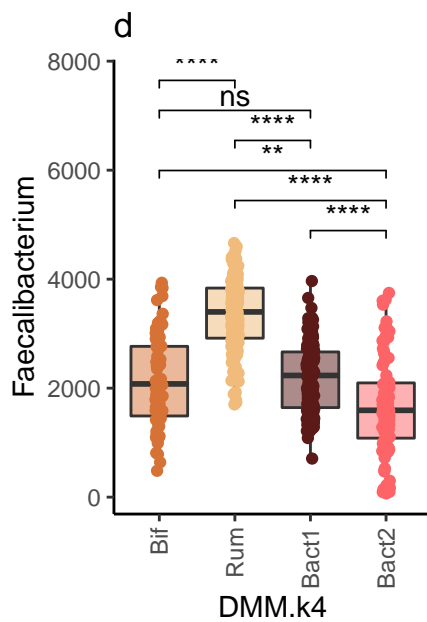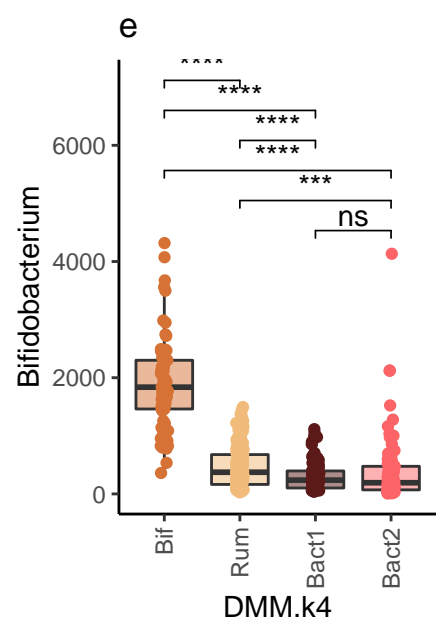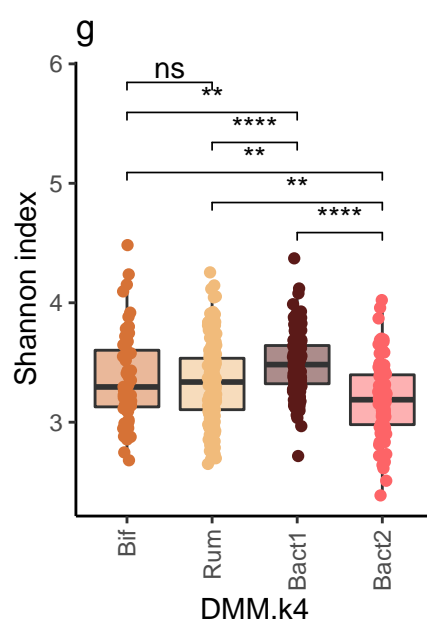

Supplement: Supplementary file 1 [file biomedicines-10-00016-s001.zip › Supplemental Figures and Table/Supplemental Figure S1.pdf]

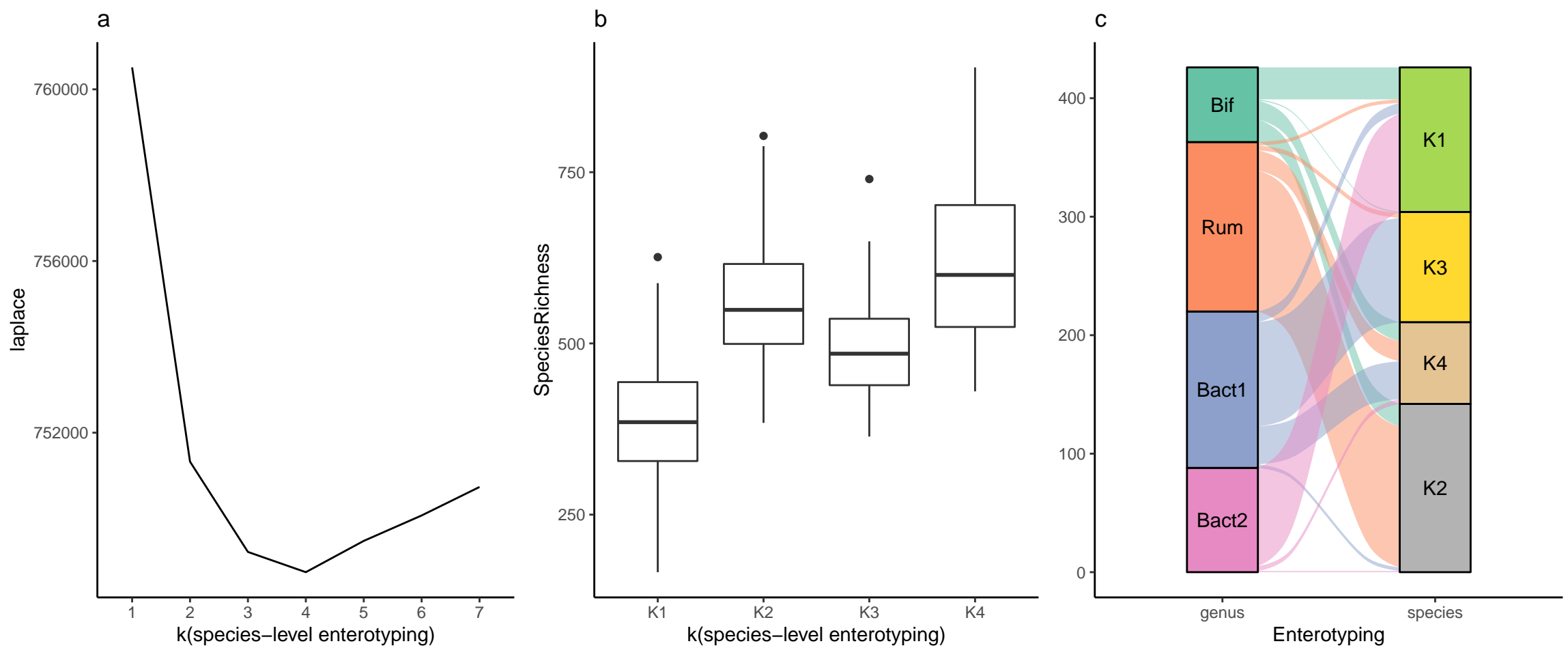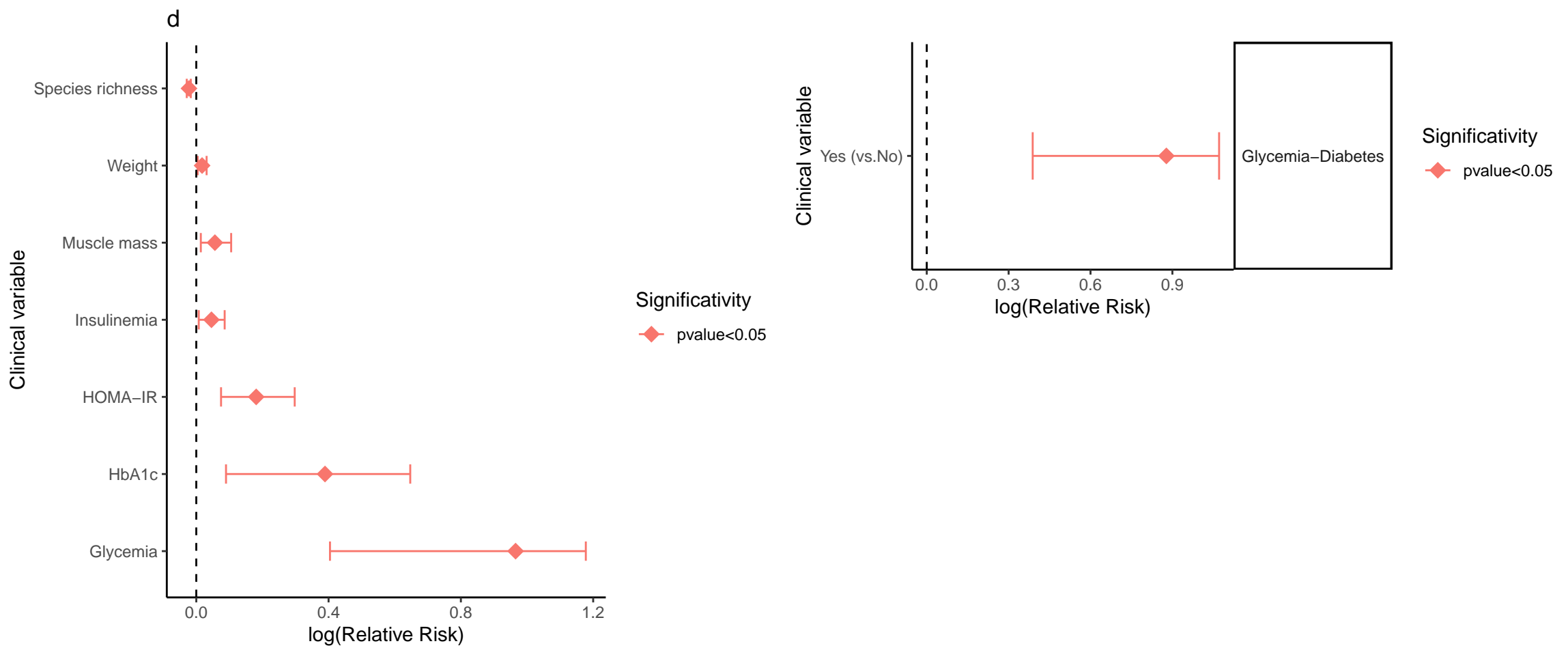

Supplement: Supplementary file 1 [file biomedicines-10-00016-s001.zip › Supplemental Figures and Table/Supplemental Figure S3.pdf]

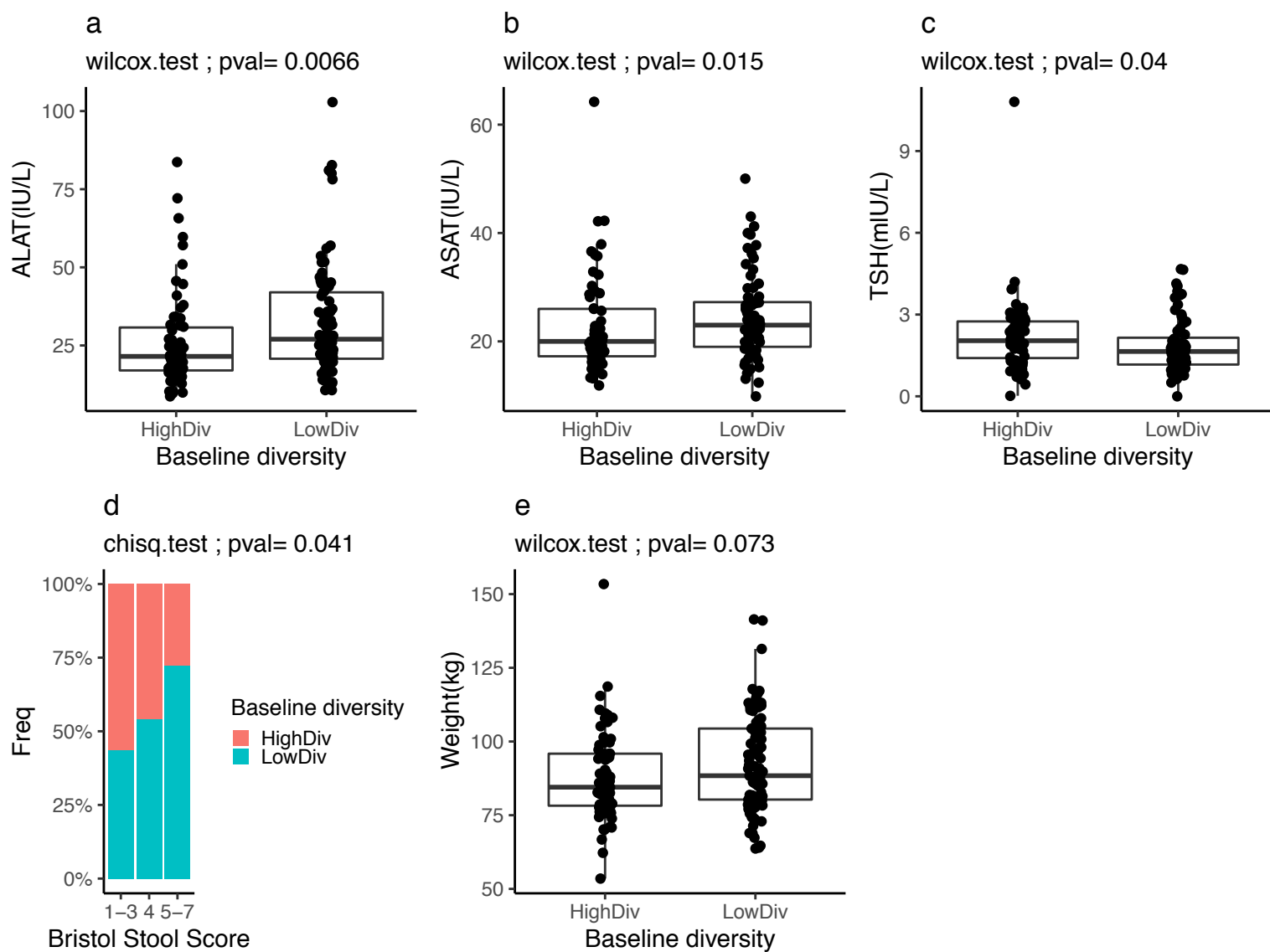

Supplement: Supplementary file 1 [file biomedicines-10-00016-s001.zip › Supplemental Figures and Table/Supplemental Figure S4.pdf]

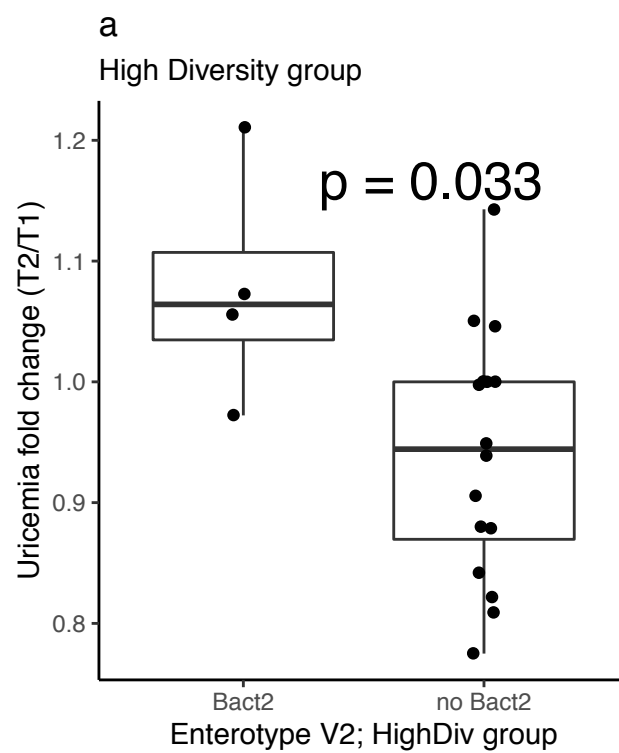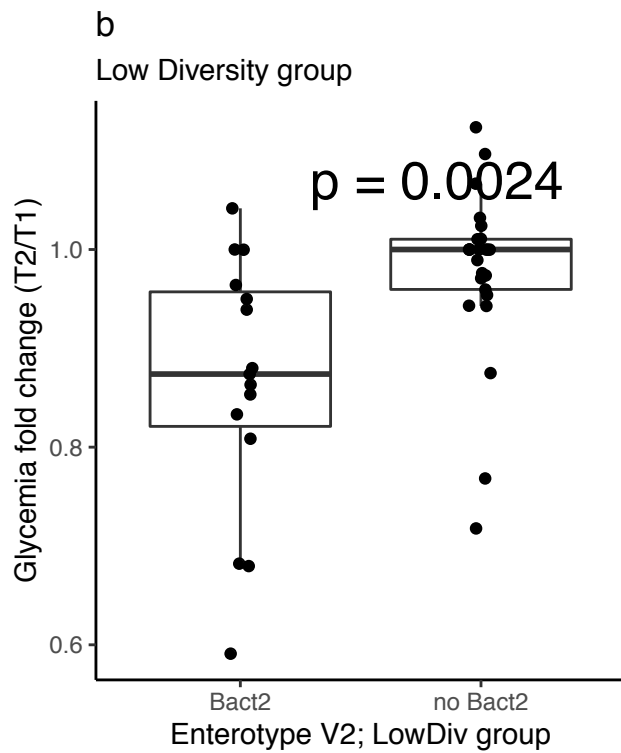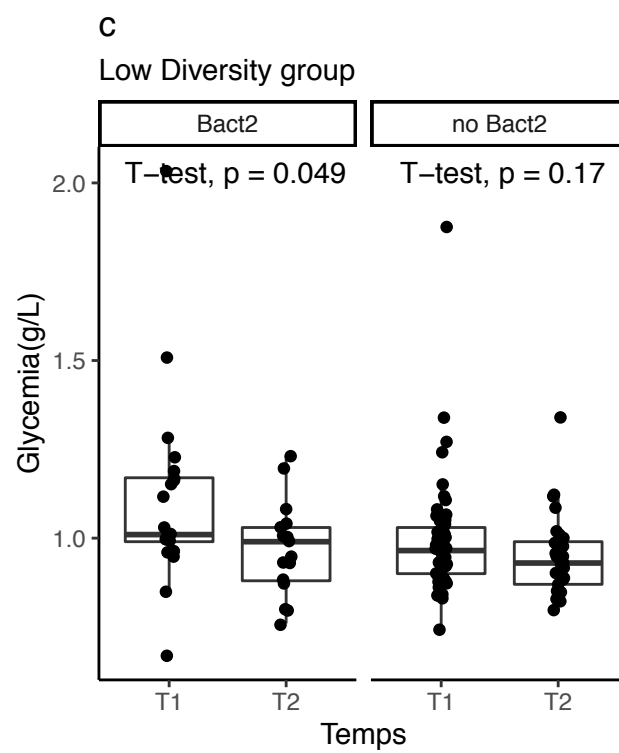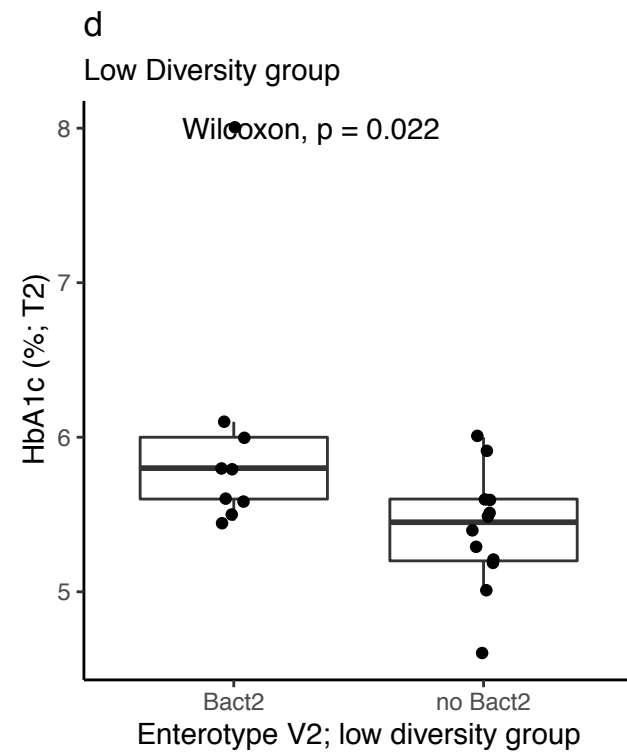

Supplement: Supplementary file 1 [file biomedicines-10-00016-s001.zip › Supplemental Figures and Table/Supplemental Figure S5.pdf]

a

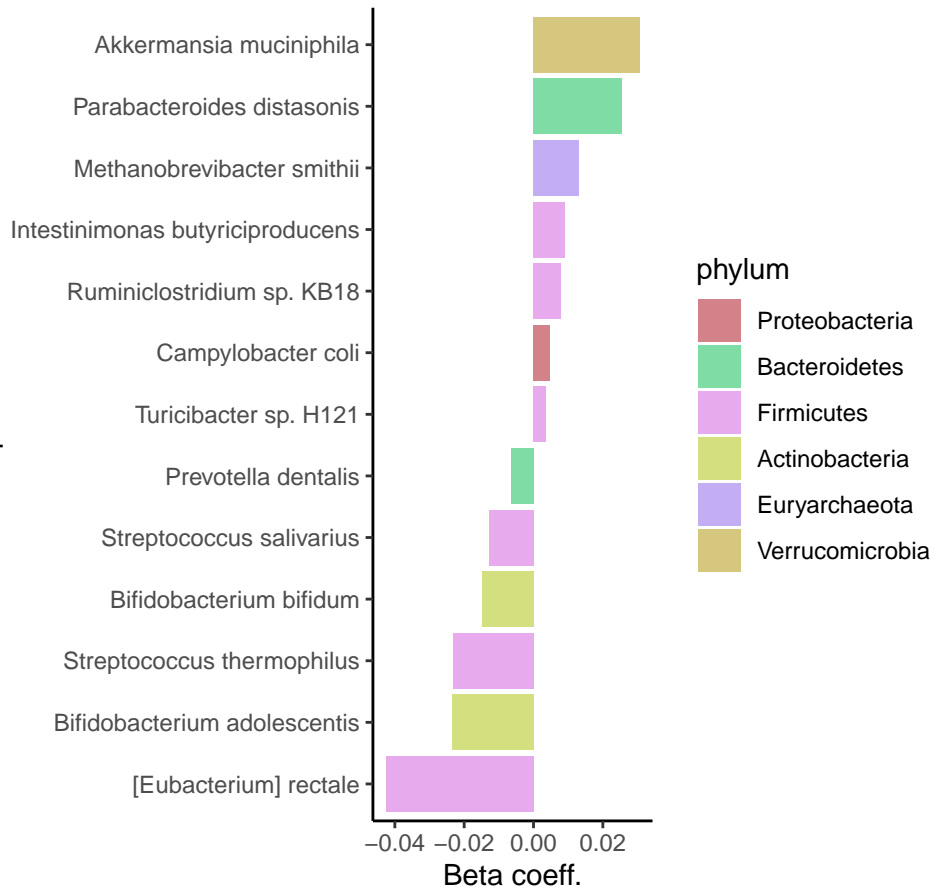

b

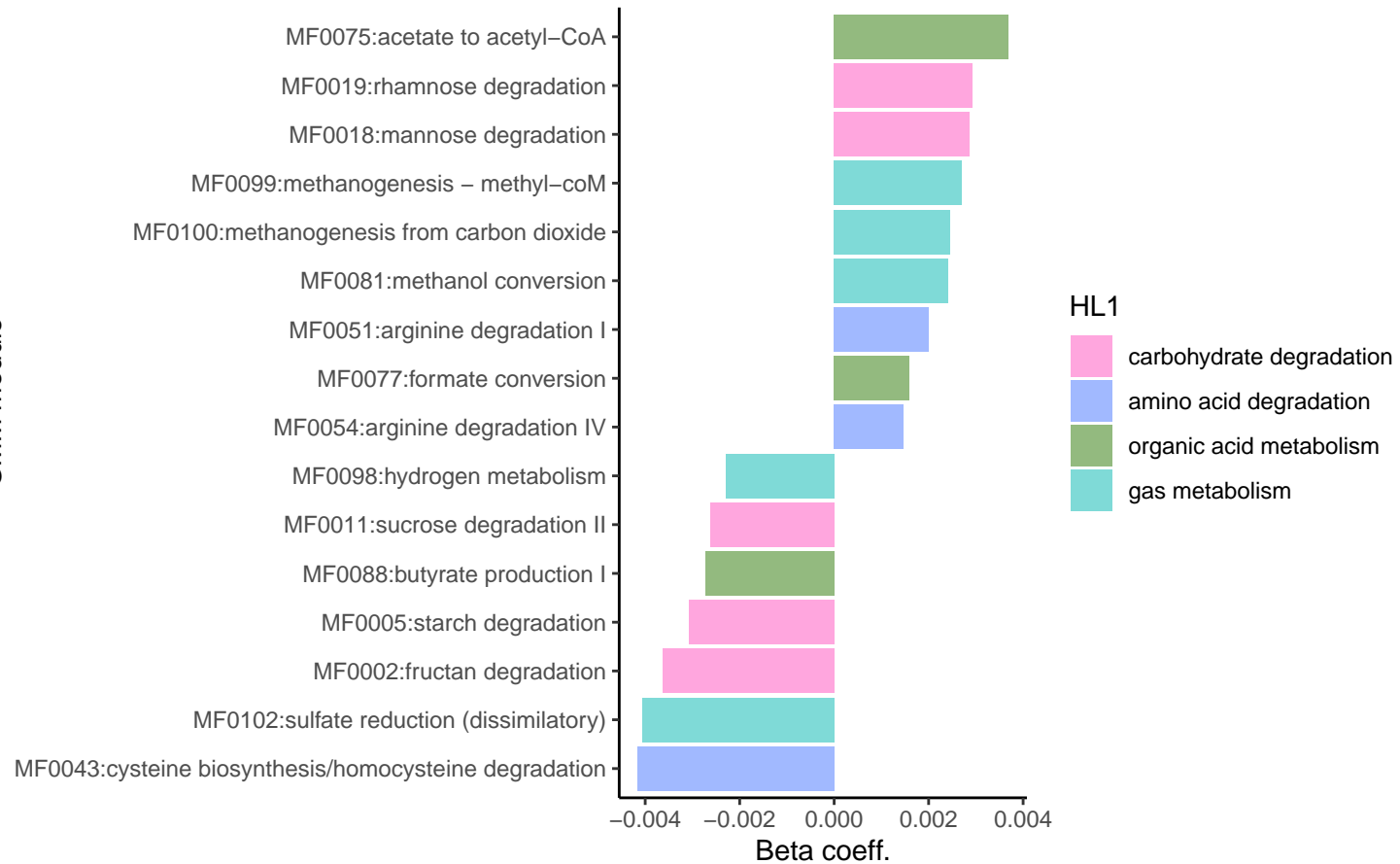

Supplement: Supplementary file 1 [file biomedicines-10-00016-s001.zip › Supplemental Figures and Table/Supplemental Figure S6.pdf]

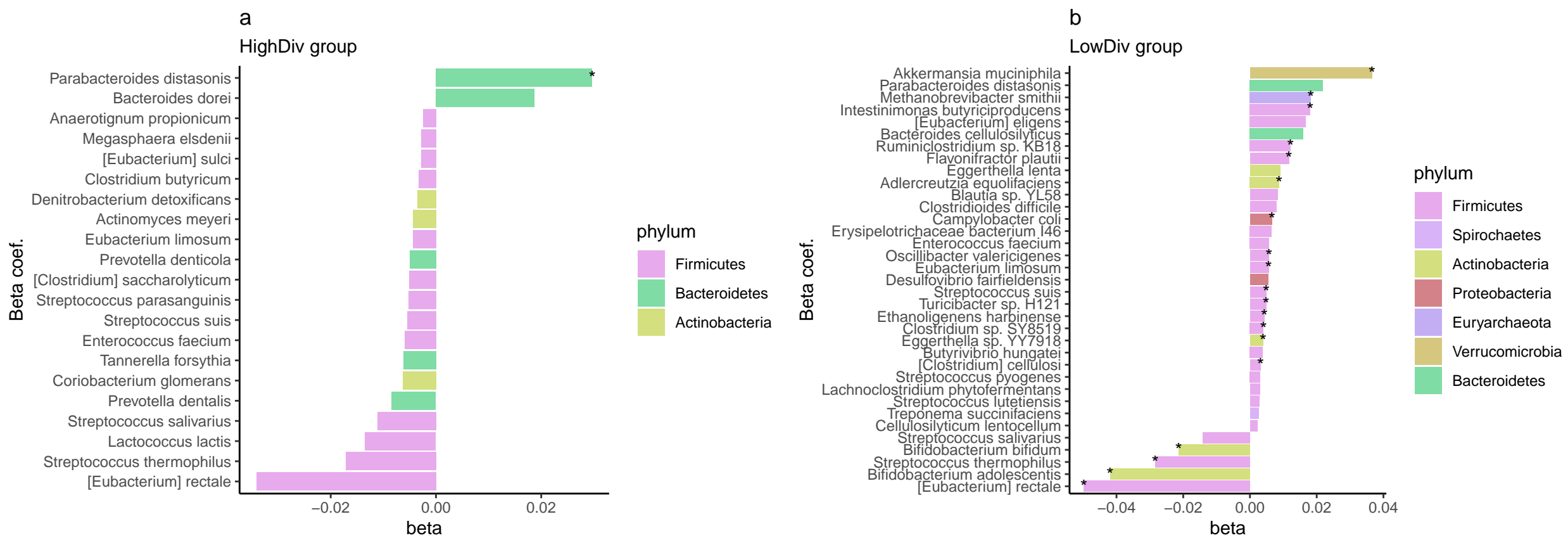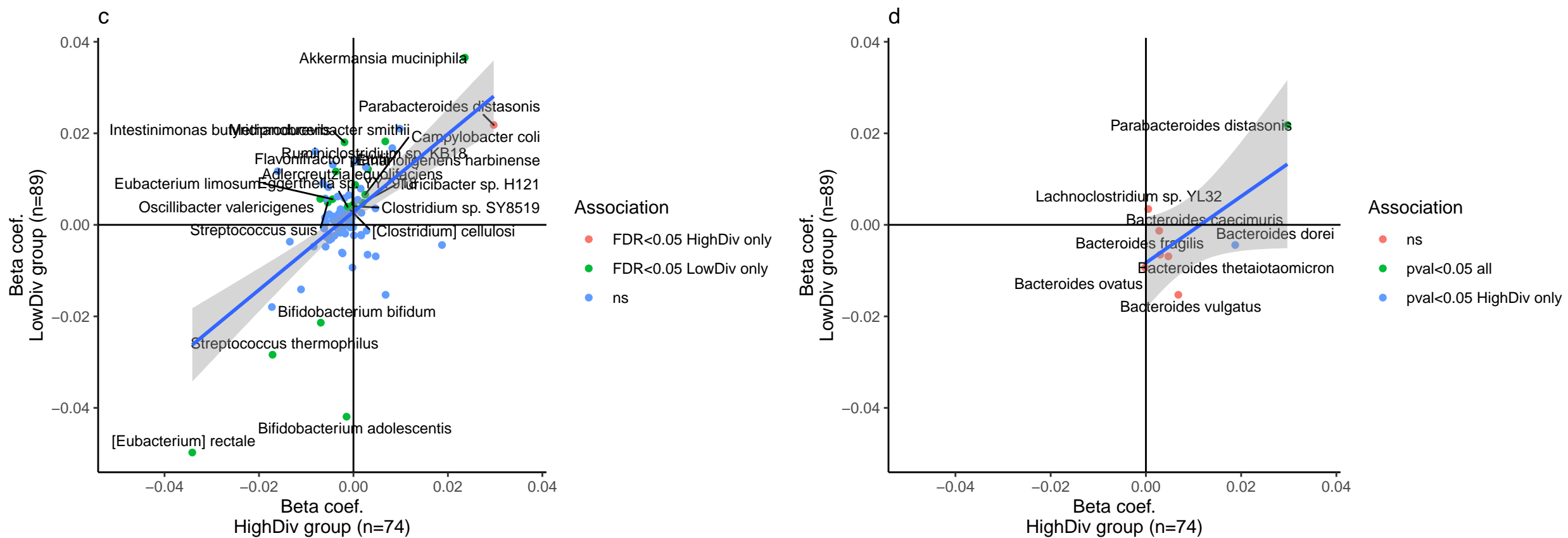

Supplement: Supplementary file 1 [file biomedicines-10-00016-s001.zip › Supplemental Figures and Table/Supplemental Figure S7.pdf]

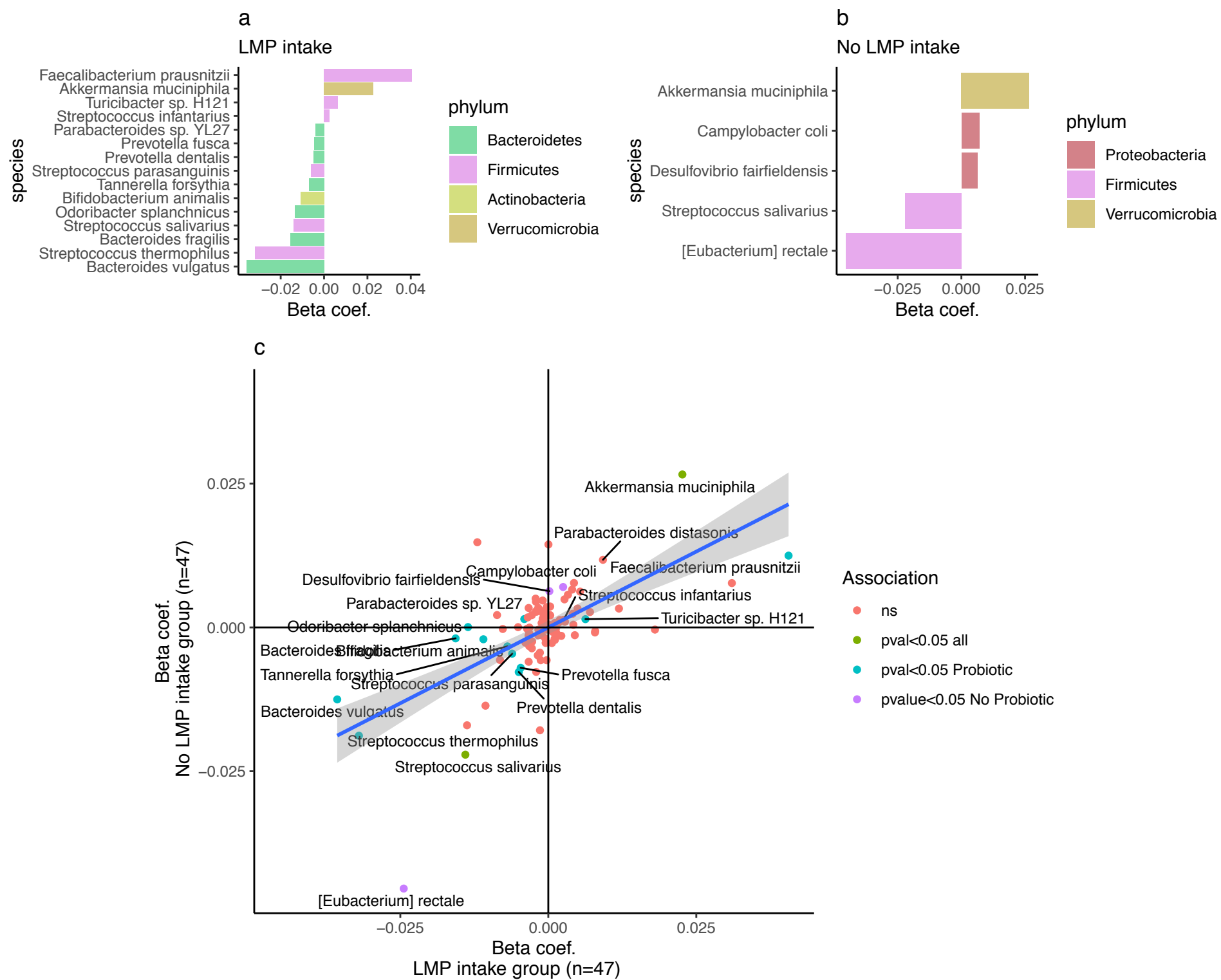

Supplement: Supplementary file 1 [file biomedicines-10-00016-s001.zip › Supplemental Figures and Table/Supplemental Figure S8.pdf]

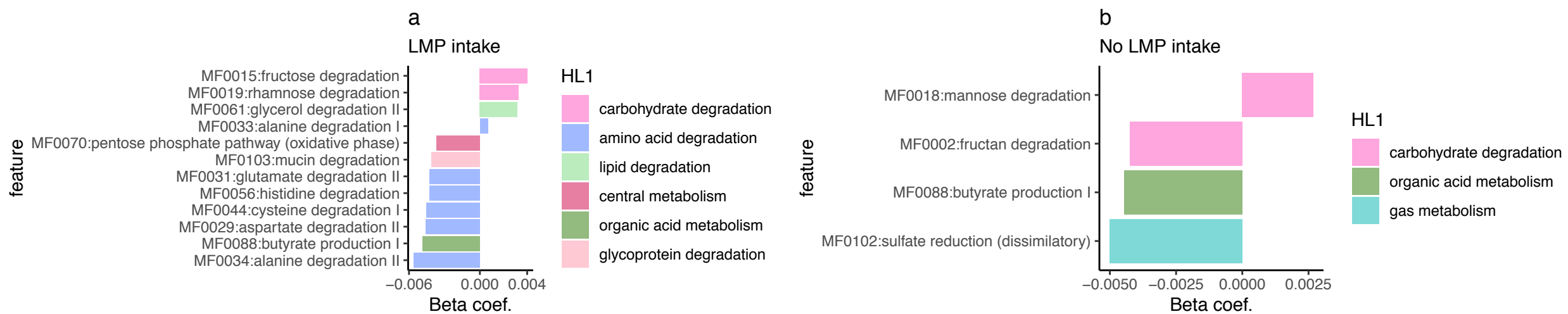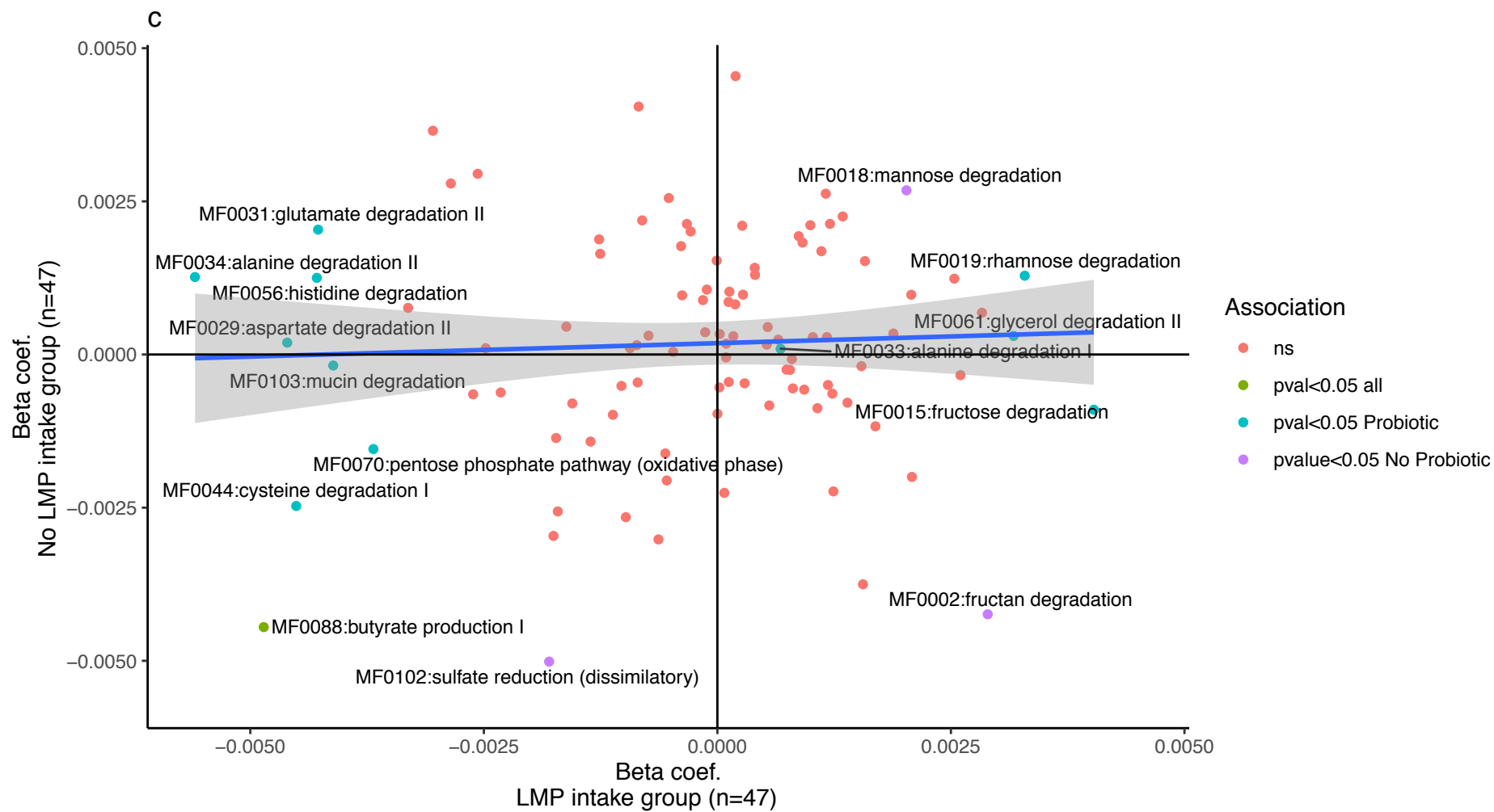

Supplement: Supplementary file 1 [file biomedicines-10-00016-s001.zip › Supplemental Figures and Table/Supplemental Figure S9.pdf]
